# Starch Solutions Prepared under Different Conditions as Modifiers of Chitosan/Poly(aspartic acid)-Based Hydrogels

**DOI:** 10.3390/ma14164443

**Published:** 2021-08-08

**Authors:** Magdalena Głąb, Anna Drabczyk, Sonia Kudłacik-Kramarczyk, Martin Duarte Guigou, Agnieszka Makara, Paweł Gajda, Josef Jampilek, Bożena Tyliszczak

**Affiliations:** 1 Department of Materials Science, Faculty of Materials Engineering and Physics, Cracow University of Technology, 37 Jana Pawła II Av., 31-864 Krakow, Poland; bozena.tyliszczak@pk.edu.pl; 2 Department of Engineering and Technology, Catholic University of Uruguay, Av. 8 de Octubre 2738, Montevideo 11600, Uruguay; martin.duarte@ucu.edu.uy; 3 Department of Chemical Technology and Environmental Analytics, Faculty of Chemical Engineering and Technology, Cracow University of Technology, 24 Warszawska St., 31-155 Krakow, Poland; agnieszka.makara@pk.edu.pl; 4 Department of Sustainable Energy Development, Faculty of Energy and Fuels, AGH University of Science and Technology, 30 Mickiewicza Av., 30-059 Krakow, Poland; pgajda@agh.edu.pl; 5 Department of Analytical Chemistry, Faculty of Natural Sciences, Comenius University, Ilkovicova 6, 84215 Bratislava, Slovakia; josef.jampilek@gmail.com; 6 Institute of Neuroimmunology, Slovak Academy of Sciences, Dubravska Cesta 9, 84510 Bratislava, Slovakia

**Keywords:** chitosan, starch, poly(aspartic acid), polysaccharides, hydrogels, tensile strength, swelling ability, simulated physiological liquids

## Abstract

Recently, there has been great interest in the application of polysaccharides in the preparation of diverse biomaterials which result from their biocompatibility, biodegradability and biological activity. In this work, the investigations on chitosan/poly(aspartic acid)-based hydrogels modified with starch were described. Firstly, a series of hydrogel matrices was prepared and investigated to characterize their swelling properties, structure via FT-IR spectroscopy, elasticity and tensile strength using the Brookfield texture analyzer as well as their impact on simulated physiological liquids. Hydrogels consisting of chitosan and poly(aspartic acid) in a 2:1 volume ratio were elastic (9% elongation), did not degrade after 30-day incubation in simulated physiological liquids, exhibited a relative biocompatibility towards these liquids and similar swelling in each absorbed medium. This hydrogel matrix was modified with starch wherein two of its form were applied—a solution obtained at an elevated temperature and a suspension obtained at room temperature. Hydrogels modified with hot starch solution showed higher sorption that unmodified materials. This was probably due to the higher starch inclusion (i.e., a larger number of hydrophilic groups able to interact with the adsorbed liquid) when this polysaccharide was given in the form of a hot solution. Hydrogels modified with a cold starch suspension had visible heterogeneous inequalities on their surfaces and this modification led to the obtainment materials with unrepeatable structures which made the analysis of their properties difficult and may have led to misleading conclusions.

## 1. Introduction

Polysaccharides as biodegradable, biocompatible and biologically active compounds belong to the group of substances with attracting growing interest in studies on the development of innovative materials for biomedical applications [1]. One of the most promising polysaccharides is chitosan, a linear biopolymer which is obtained as a result of the deacetylation of the other polysaccharide—chitin. Chitosan shows properties such as antimicrobial activity, bioactivity and enzymatic biodegradability [2]. The application of this polysaccharide is widely considered for tissue regeneration. For example, Smirnova et al. developed polymer nanocomposites consisting of chitosan and chitin nanofibrils. It was demonstrated that the adequate content of chitin nanofibrils—5 wt. %—resulted in the preparation of materials with optimal physicochemical and mechanical properties as well as showing bioactivity to human dermal fibroblasts [3]. In turn, Jiang et al. prepared chitosan-based scaffolds with specially designed architecture of microchannels. The development of systems with controllable density, diameter and arrangement of channels led to obtaining materials which promoted proliferation of cells and their in vitro distribution. Moreover, it was proved that such systems positively affected the in vivo ingrowth of cells and tissues as well as in vivo vascular formation [4]. Numerous investigations were performed to evaluate properties and potential of chitosan as a main component of scaffolds for tissue regeneration [5,6,7,8].

Due to chitosan possessing many functional groups in its chemical structure it is possible to use this polysaccharide for preparation of carriers for various active substances which may form bonds with the mentioned groups [9]. Chitosan-based carriers were investigated as systems for delivery of many anticancer [10,11,12] and anti-inflammatory drugs [13,14]. Among many applications of chitosan for biomedical purposes, one of the most promising ones seems to be its use for preparation of modern wound dressing materials [15]. For example, film-forming emulsions consisting of chitosan and selected essential oils were tested as wound-healing materials by Pereira dos Santos et al. [16]. Importantly, many studies were conducted to develop multifunctional chitosan-based dressings with antibacterial properties and with an ability to controlled drug release [17,18,19,20].

Another polysaccharide widely considered for biomedical applications is starch. There is growing interest from scientists is in its application for the development of innovative dressing materials. For example, hydrogel dressings based on a combination of itaconic acid, starch and Fe_3_O_4_ nanoparticles were investigated by Nezami et. al. They proved that the developed polymers were pH-responsive materials able to control the release of guaifenesin [21]. In turn, starch-based nano-fibrous scaffolds with wound healing application potential were tested by Hadisi et al. [22] and Waghmare et al. [23] while Delavari and Stiharu developed composite films based on starch and poly(vinyl alcohol) with the same application potential [24].

Considering their properties, a material containing both chitosan and starch could have potential for biomedical use. Hydrogel dressings based on starch, chitosan and poly(vinyl alcohol) containing additionally nano zinc oxide showing antibacterial activity were developed by Baghaie et al. [25]. Next, nanofibrous mats based on the same components but prepared via electrospinning were analyzed by Adeli et al. [26]. Modern wound dressings containing both chitosan and starch were also evaluated by Alborzi et al. [27] and Su et al. [28].

In this work, a synthesis methodology and investigations on chitosan/poly(aspartic acid)-based hydrogels modified with starch are presented. Firstly, a series of investigations was performed to select a hydrogel matrix consisting of both above-mentioned reagents in an appropriate volume ratio. For this purpose, the analysis of swelling properties, elasticity and tensile strength, interactions with simulated physiological liquids as well as characterization of the chemical structure via Fourier transform infrared (FT-IR) spectroscopy of obtained hydrogel matrices were conducted to determine the matrix with the most beneficial properties. Subsequently, selected polymer matrix was modified with starch wherein its two forms were used—a solution prepared at high temperature and a suspension obtained at room temperature. Chitosan/poly(aspartic acid)-based hydrogels modified with starch were investigated including the analysis of their surface morphology, chemical structure, mechanical properties (elasticity and tensile strength) and sorption capacity. The main purpose of the research was to evaluate the usefulness of obtained starch-modified hydrogels for biomedical applications.

## 2. Materials and Methods

### 2.1. Materials

Chitosan (low molecular weight 50,000–190,000 Da; deacetylation degree 75–85%), diacrylate poly(ethylene glycol) (crosslinker, Mn = 700 g/mol), 2-hydroxy-2-methylpropiophenone (photoinitiator, d = 1.077 g/mol, 97%, Mw = 164.2 g/mol) and L-aspartic acid (≥98%) were purchased in Merck (Darmstadt, Germany). Starch (natural potato-starch) was bought in Avantor Performance Materials Poland S.A. (Gliwice, Poland). All reagents were applied without further purification.

### 2.2. Synthesis of Chitosan/Poly(aspartic acid)-Based Hydrogels

Chitosan/poly(aspartic acid)-based hydrogels were obtained via the photopolymerization process. This method was selected because it allows to obtain materials in a short time and there is no need to use any toxic organic solvents which is significant in terms of potential application of designed materials for biomedical purposes. Here, one of the most important parameters is an adequate proportion of applied reagents (particularly a crosslinker and a photoinitiator). However, this may be verified, e.g., during incubation studies where uncrosslinked reagents may affect pH of incubation liquids.

Firstly, 1% chitosan solution in 0.05% acetic acid and 0.5% aqueous solution of aspartic acid were prepared. Next, adequate amounts of these solutions were mixed with crosslinker and photoinitiator and such prepared mixtures were treated with UV radiation for 2 min. A VP-60 EMITA lamp was used as a source of radiation (180 W, λ = 320 nm, Famed, Lodz, Poland). Compositions of obtained hydrogels are presented in Table 1.

Prepared hydrogels were next dried at room temperature and samples were selected for modification with starch. The studies performed included the evaluation of sorption properties of obtained polymers, determining their behavior in selected liquids, assessment of their mechanical properties and characteristics of their chemical structure. The hydrogel sample with the most favorable properties was subsequently subjected to the modification process.

### 2.3. Synthesis of Chitosan/Poly(aspartic acid)-Based Hydrogels Modified with Starch

Starch-modified hydrogels were obtained analogously to the unmodified materials. Briefly, 1% chitosan solution was mixed with 0.5% aspartic acid solution (one hydrogel matrix was selected from Table 1 based on the performed investigations), crosslinker and photoinitiator. Additionally, starch suspension was added to the reaction mixture wherein two types of suspensions were applied, i.e., an aqueous starch suspension obtained at room temperature (defined as “cold starch”) and an aqueous suspension of this polysaccharide obtained at 60 °C (defined as “hot starch”). The whole reaction mixture containing starch was intensively stirred before the UV treatment. The photopolymerization process was performed for 2 min using the same lamp as previously. Compositions of obtained starch-modified hydrogels are presented in Table 2.

**Figure 1:**
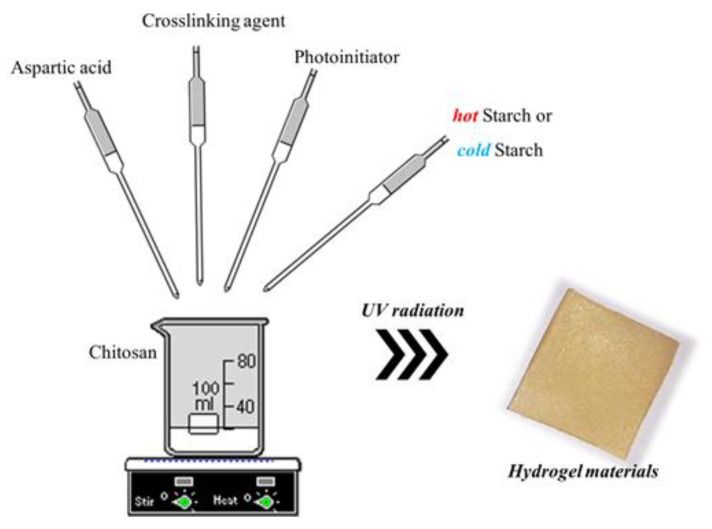
The scheme of the synthesis of hydrogels.

The scheme of the synthesis of hydrogel materials is shown in Figure 1.

**Figure 2:**
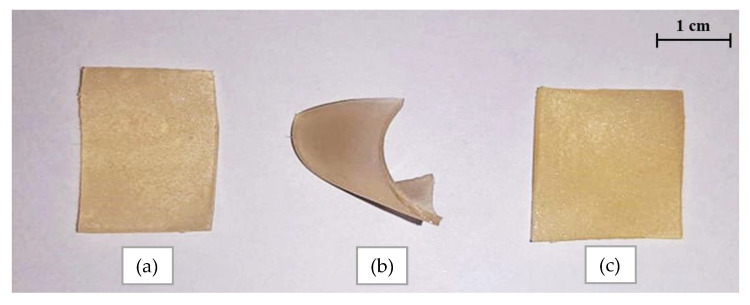
Images of hydrogels: unmodified sample (**a**), sample modified with cold starch suspension (**b**) and hot starch solution (**c**).

Next, obtained hydrogels containing starch were dried at room temperature. Below, in Figure 2, the images of dried samples are presented.

As can be noticed in Figure 2, hydrogel sample modified with cold starch suspension curled up during drying which may suggest that this sample showed worse mechanical properties than the remining samples. The observed sample arrangement may be caused by heterogeneous distribution of starch particles in the matrix of such modified material.

Prepared hydrogels were subsequently investigated in terms of determining their swelling ability and mechanical properties. Furthermore, morphology of the polymers as well as presence of functional groups in their structure were characterized. Attention was paid to the impact of the modification of hydrogels with starch on their properties. Importantly, the dependence between these properties and the type of the starch suspension introduced into the hydrogel matrices was also verified.

### 2.4. Applied Methodology

#### 2.4.1. Characterization of the Sorption Properties of Hydrogels

Developed hydrogels were considered for biomedical applications including wound dressing materials. Thus, their swelling properties were evaluated in terms of the potential capability of the wound exudate sorption by such dressings. When the dressing material is able to absorb the resulting exudate, the wound healing may proceed faster hence the importance of such a study in view of the potential use of the tested materials.

The research was performed using selected simulated physiological liquids including hemoglobin (1% aqueous solution), SBF (simulated body fluid, isotonic to the human blood plasma [29]), Ringer liquid (infusion liquid) and distilled water (as a reference liquid). Firstly, approximately 1.0 g of each hydrogel sample was introduced into the 50 mL of tested liquid. After 1 h, samples were separated from the liquids, weighed in a swollen state and immersed again. The whole procedure was repeated after 24 h and 72 h. The swelling ability of hydrogels was determined using the swelling ratios *Q* which were calculated by the following Equation (1):

(1) $$Q=\frac{m-m_{0}}{m_{0}}$$

where: *Q*—swelling ratio, g/g; *m*—weight of sample after swelling, g; *m*_0_—weight of sample before swelling, g.

The results were presented in a form of bar charts showing the swelling ratio calculated for each tested hydrogel sample depending on the type of the liquid absorbed and the sorption time.

The study was performed both for unmodified and modified hydrogels. The analysis of swelling sorption of unmodified polymers aimed at determining the impact of the composition of hydrogel matrix on this property. In turn, swelling tests of hydrogels modified with starch were conducted to evaluate whether the polysaccharide introduced into the polymer matrix affected the sorption capability of such modified materials.

#### 2.4.2. Assessment of the Behavior of Chitosan/Poly(aspartic acid)-Based Hydrogels in the Environment of Simulated Physiological Liquids

Evaluation of the behavior of obtained hydrogels in environments simulating selected physiological liquids is important in terms of their potential biomedical application. Such a study included the immersion of hydrogel samples for 30 days in 50 mL of distilled water, SBF, hemoglobin and Ringer liquid (the same liquids in which the swelling ability was determined). The incubation was performed at 36.7 °C wherein such conditions were applied to simulate the temperature of human body. The study involved the pH measurements of liquids with incubated samples made using the pH-meter Elmetron CX-701 (Zabrze, Poland). Analysis was performed for chitosan/poly(aspartic acid)-based hydrogels.

#### 2.4.3. Characteristics of the Chemical Structure of Hydrogels Using FT-IR Spectroscopy

Fourier transform infrared (FT-IR) spectroscopy was applied to verify the presence of functional groups in the structure of both unmodified and modified hydrogel polymers. Importantly, samples of chitosan/poly(aspartic acid)-based hydrogels after incubation studies in simulated physiological liquids were also subjected to the FT-IR technique to determine the impact of these environments on the structure of tested polymers. The analysis was conducted at room temperature via the Thermo Scientific Nicolet iS5 equipped additionally with ATR diamond accessory (Loughborough, UK). The FT-IR spectra of analyzed samples were recorded within the range of 4000–500 cm^−1^ (32 scans, resolution 4.0 cm^−1^).

#### 2.4.4. Evaluation of the Surface Morphology of Hydrogels via Scanning Electron Microscopy (SEM)

Subsequently, the surface morphology of both unmodified hydrogels and polymers containing starch was characterized. The analysis was performed using the Jeol 5510LV Scanning Electron Microscope (Jeol Ltd., Tokyo, Japan). Before the analysis, hydrogel samples were dried at room temperature and sputtered with gold. The study was performed at room temperature.

#### 2.4.5. Assessment of the Mechanical Properties of Hydrogel Materials

Studies on the mechanical properties of hydrogels are significant because they may provide information on such parameters of the tested polymers as their elasticity or tensile strength. In turn, the knowledge of such properties allows proper evaluation of the suitability of the materials under specific conditions of use. Studies were performed by means of the Brookfield CT3 texture analyzer (Middleboro, MA, USA) according to the ISO 37 type 2 and ISO 527-2 type 5A standards. Firstly, polymers after the synthesis were treated using the ZCP020 Manual Cutting Press to prepare paddle-shaped hydrogel samples (dimensions: depth—1.5 mm, width—3.0 mm, length—30.0 mm) which were subsequently dried at room temperature under pressure to keep their shape and to avoid wrinkling. In order to perform the measurements, paddle-shaped hydrogels were fixed between the jaws of the texture analyzer using clamps. When the measurement started, the jaws parted which resulted in the samples’ stretching. The measurement was carried out until the hydrogel ruptured.

The analysis was performed both for unmodified hydrogels and hydrogels containing starch. The investigations aimed at determining the impact of the composition of the hydrogel matrices on their mechanical properties as well as to determine whether the starch introduced into the polymer affected these properties.

## 3. Results and Discussion

### 3.1. Results of Studies on the Swelling Properties of Hydrogels

**Figure 3:**
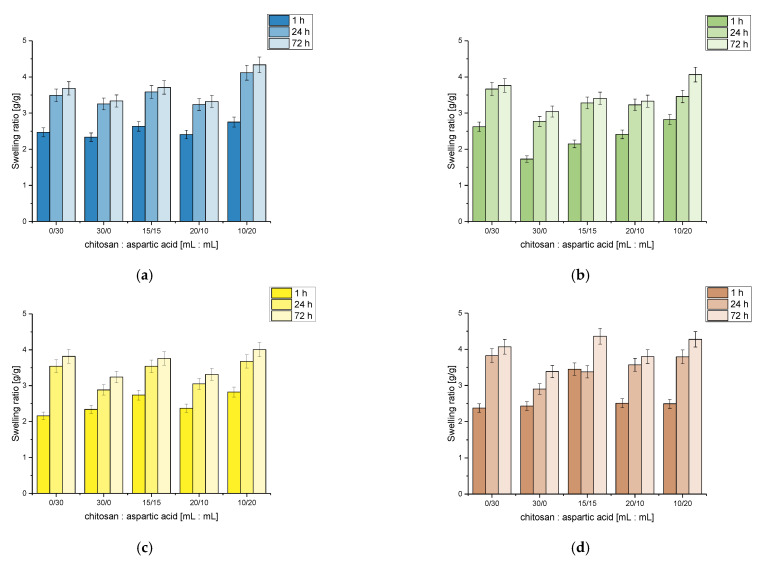
Results of swelling studies of hydrogels in distilled water (**a**), Ringer liquid (**b**), SBF (**c**) and hemoglobin (**d**).

Results of the investigations on the swelling ability of chitosan/poly(aspartic acid)-based hydrogels in selected simulated physiological liquids are presented below in Figure 3.

As it may be noticed considering above-presented results, the volume ratio of the reagents used during the synthesis of hydrogel matrices affected the swelling properties of tested hydrogels. It was observed that the sample obtained using 10 mL of chitosan solution and 20 mL of aspartic acid solution showed the highest swelling ratio in tested liquids. In turn, the lowest swelling ability was reported for samples 30/0 and 20/10, i.e., for hydrogels prepared using 0 mL and 10 mL of aspartic acid solution, respectively. Thus it may be stated that the amount of the aspartic acid in hydrogel matrix affected the sorption of analyzed materials. The larger amount of this reagent in the polymer matrix, the better swelling properties of hydrogels. Considering the swelling ability of all tested hydrogels, it may be concluded that a larger amount of aspartic acid compared to the amount of chitosan in the analyzed polymer matrices caused an increase in their sorption capacity. This was probably caused by the chemical structure of aspartic acid which contains many hydrophilic groups resulting in larger sorption of liquids. Thus the larger the amount of this acid in the hydrogel matrix, the better sorption properties of such obtained materials. For example, Suhail et al. reported that an increase in the content of aspartic acid in swelled polymers leads to an increase in a hydrophilicity of hydrogel network and thus to its higher swelling ability [30].

Analyzing the results obtained in all tested liquids, it was observed that the lowest swelling properties were reported for Ringer liquid. It may be caused by the presence of various (divalent) ions in this solution. They contributed to the increase in the crosslinking degree of swelled hydrogel which, in turn, led to the decrease in its swelling ability. Additional crosslinks may form between the polymer chains which may result in the decrease in the volume of free spaces in the polymer structure which is available for absorbed liquid. On the other hand, the highest sorption capacity was observed in hemoglobin. Lack of additional ions in this liquid meant that any additional crosslinks were not formed, and in turn, the liquid freely penetrated the hydrogel structure resulting in an increase in its sorption capability.

Considering graphs showing the results of swelling investigations of obtained hydrogels it may be noted that the most beneficial properties in terms of the future application of developed materials for biomedical purposes showed sample 20/10 (obtained using 20 mL of chitosan solution and 10 mL of aspartic acid solution). This hydrogel matrix exhibited similar swelling properties in each tested liquid. Such a behavior is beneficial for use in medicine and pharmacy because the material obtained using the mentioned amounts of the reagents will behave in a similar manner in each environment occurring in the human body. Importantly, this sample did not show averaged values of swelling ratio among all tested hydrogels therefore such a prepared matrix exhibited a potential for a wide range of applications in medicine and pharmacy. When applying such a material, its desired property is to absorb as much liquid as possible. During the application of the tested materials in cosmetics, e.g., in a form of moisturizing eye pads, a large sorption capacity of such applied hydrogels is not beneficial because it might cause the skin to dry out and the swelled material might become too heavy and thus uncomfortable.

### 3.2. Results of pH Measurements Determined during the Incubation of Obtained Hydrogels in Selected Simulated Physiological Liquids

**Figure 4:**
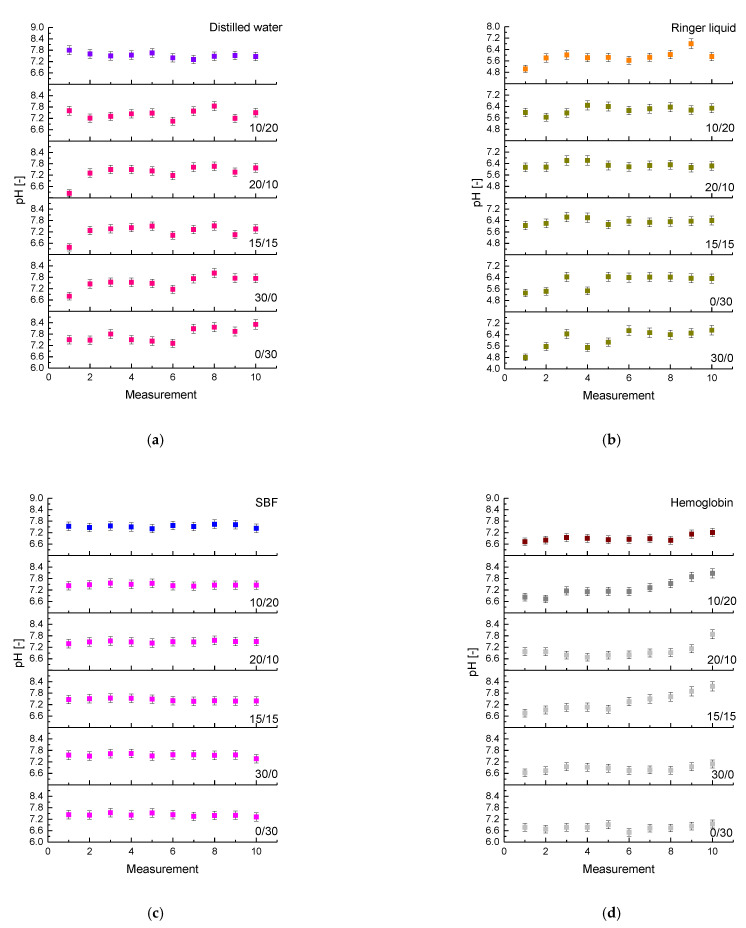
Results of incubation studies in distilled water (**a**), Ringer liquid (**b**), SBF (**c**) and hemoglobin (**d**).

Changes in pH of the solutions during the 30-day incubation of hydrogels are shown below in Figure 4. Results for each incubation liquid are presented separately wherein the measurements were also conducted for incubation liquids without hydrogel samples (as reference measurements).

Analyzing above-presented results, it may be observed that in the case of all immersed samples pH values of liquids measured during the incubation were only slightly different. The buffering properties of the analyzed hydrogels were most visible in the SBF solution thus such an environment seemed to be the most adequate for further studies on the developed matrices in terms of their use for biomedical purposes.

Performed incubation investigations allowed to indicate that the most promising results were observed for sample 20/10 (obtained using 20 mL of chitosan solution and 10 mL of aspartic acid solution). This matrix showed a relative biocompatibility towards each tested simulated physiological liquid. In pH values measured during the incubation of this sample maintained at the same level, only slight deviations were observed. pH changes observed during the whole period of the incubation of sample 20/10 seemed to be the lowest which, in turn, indicated good buffering properties of this polymer and its good stability in tested simulated physiological liquids.

### 3.3. Characterization of the Chemical Structure of Hydrogels via FT-IR Technique

**Figure 5:**
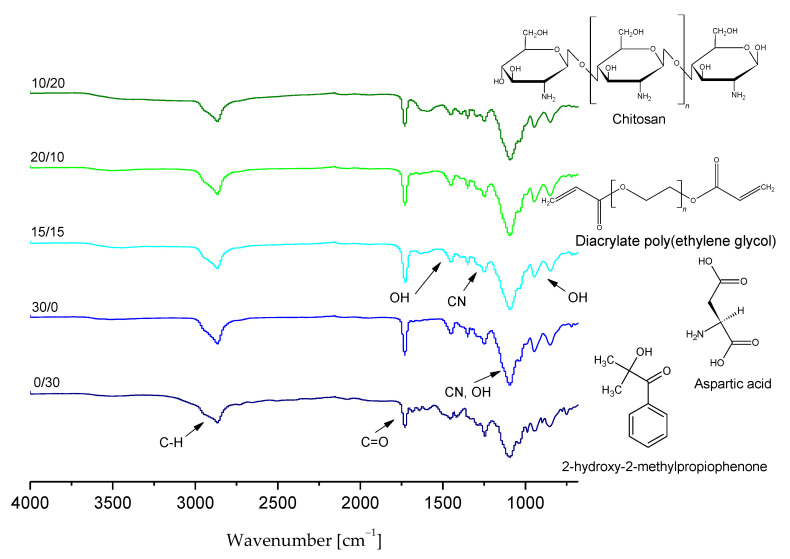
FT-IR spectra of chitosan/poly(aspartic acid)-based hydrogels.

The presence of functional groups in the hydrogels’ structure was verified using FT-IR spectroscopy. Obtained FT-IR spectra of chitosan/poly(aspartic acid)-based polymer matrices are presented in Figure 5.

**Figure 6:**
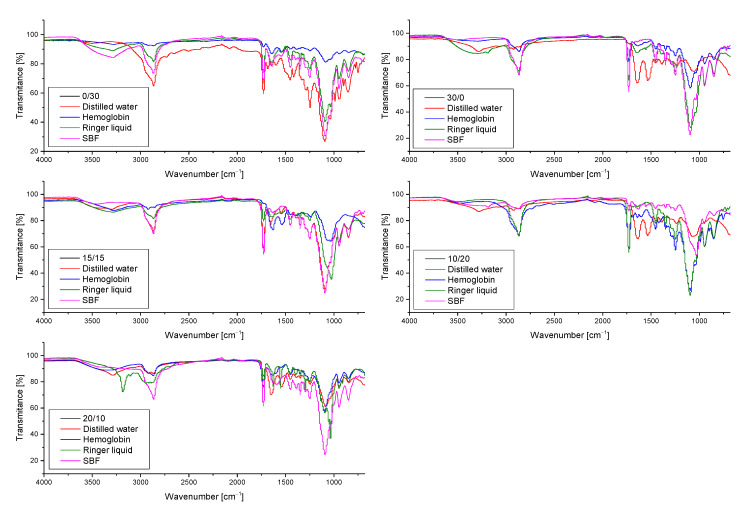
FT-IR spectra showing the impact of the incubation studies on the structure of unmodified chitosan/poly(aspartic acid)-based hydrogel samples.

Furthermore, hydrogel samples after incubation studies were also subjected to the FT-IR analysis to determine the impact of such an immersion on their structure. The resulting FT-IR spectra of samples both before and after incubation in tested liquids are shown in Figure 6.

Based on the above-presented FT-IR spectra it was possible to identify the functional groups occurring in tested hydrogels as well as to check the impact of their 30-day incubation in simulated physiological liquids on the chemical structure of these polymers and their potential degradation. On obtained FT-IR spectra of all analyzed samples wide bands at 3000–2970 cm^−1^ corresponding to C–H group were observed. They probably derived from the photoinitiator applied, which has an aromatic ring in its chemical structure. Next, in the case of all samples bands within the range 3300–3250 cm^−1^ were also noticed which corresponded to OH groups of hydrogel matrices consisting of chitosan and aspartic acid. Moreover, bands within the range 1750–1730 cm^−1^ corresponding to carbonyl group as well as bands at 1100–1000 cm^−1^, probably derived from chitosan, were also reported.

It was proved that on the FT-IR spectra of both samples before and after incubation in simulated physiological liquids bands corresponding to the same functional groups were indicated. The bands observed differed only in the intensity. In the case of FT-IR spectrum of sample 0/30 after incubation in hemoglobin a significant decrease in the intensity of the majority of bands (apart from the band corresponding to the distilled water which overlapped probably with the band deriving from the sample before the modification) was observed. It may be caused by the partial degradation of incubated materials.

Based on the spectroscopic analysis it was concluded that sample 20/10 showed the most beneficial properties compared to the other tested ones. This polymer matrix did not degrade during 30-day incubation in various simulated physiological liquids and did not show any structural changes as a result of such an incubation. Furthermore, it was noticed that the differences between the intensities of bands visible on the FT-IR spectra of this sample before and after incubation were the slightest compared to the differences observed on the FT-IR spectra of the other tested samples.

### 3.4. Results of the Investigations on the Tensile Strength of Hydrogels

**Figure 7:**
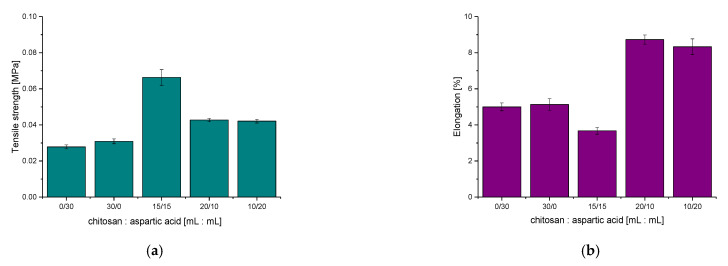
Results of mechanical studies showing the tensile strength (**a**) and the elongation (**b**) of hydrogel matrices under the tension applied.

Subsequent analysis involved determining selected mechanical parameters of obtained chitosan/poly(aspartic acid)-based hydrogel matrices. Results of performed investigations are presented below in Figure 7.

Based on the results of performed mechanical investigations, the dependence between the elasticity of tested hydrogels and their chemical compositions may be clearly observed. The lowest tensile strength was reported for samples which consisted only of one reagent, i.e., chitosan (sample 30/0) or aspartic acid (sample 0/30). The hydrogel sample containing the same amounts of these reagents also did not show satisfactory properties—it exhibited the biggest tensile strength wherein its elasticity was the lowest compared to the other tested samples.

Sample 20/10 was selected for further investigations because this hydrogel showed the most beneficial mechanical properties among all tested samples. This material was characterized by the highest elasticity, did not deform during the analysis and exhibited appropriate tensile strength. In terms of the application of the developed materials for biomedical purposes such properties are highly desirable. Mechanical investigations are important in view of the potential biomedical uses—e.g., the elasticity of such materials is significant in the case of their application as wound dressings applicable on wounds placed in sites with high mobility.

### 3.5. Results of Investigations on the Sorption Properties of Hydrogels Modified with Starch

**Figure 8:**
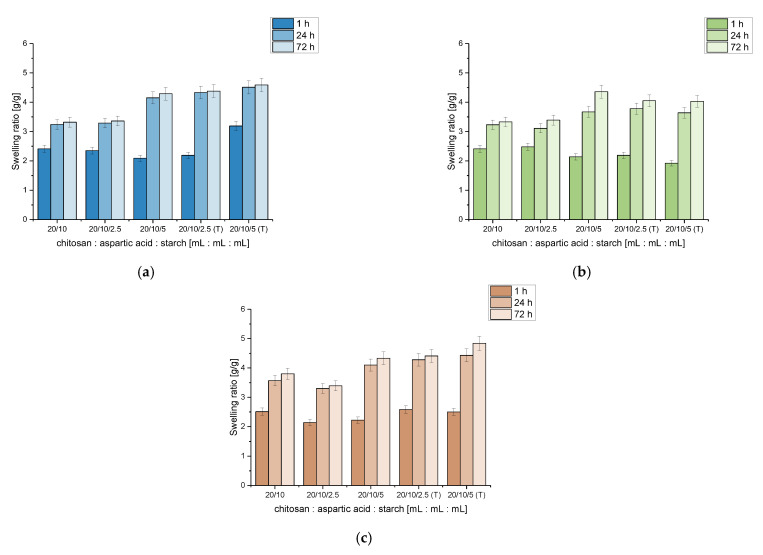
Results of sorption of hydrogels modified with starch in distilled water (**a**), Ringer liquid (**b**) and hemoglobin (**c**).

Swelling properties of chitosan/poly(aspartic acid)-based hydrogels modified with starch were also investigated to evaluate the impact of this polysaccharide on hydrogels’ sorption ability. Results of performed analyses are shown below in Figure 8.

Verifying the results obtained as a result of studies on swelling properties of chitosan/poly(aspartic acid)-based hydrogels modified with starch it was observed that hydrogel polymers containing this polysaccharide showed better swelling properties after 72 h of the study compared to the unmodified samples. This result from the fact that the introduction of starch into the hydrogel matrix enhanced its hydrophilicity (starch possesses numerous hydrophilic groups in its chemical structure) which was also proved by Parvathy et al. [31] and Ismail et al. [32]. The conclusion that the starch increases the ability of materials modified with this polysaccharide to absorb larger amount of liquids was also reported by Almeida et al. [33].

For example, the swelling ratio of unmodified hydrogel (sample 20/10) after 72 h sorption in distilled water was 3.32 g/g while this parameter in the same swelling conditions for hydrogel sample modified with 5.0 mL of “cold starch” was 4.29 g/g and 4.59 g/g for hydrogel containing 5.0 mL of “hot starch”. The same was observed for 72 h swelling in Ringer liquid, i.e., 3.33 g/g for unmodified hydrogel, 4.36 g/g for sample with 5.0 mL of “cold starch” and 4.03 g/g for sample with 5.0 mL “hot starch”, respectively.

Only samples modified with “cold starch” (2.5 mL) did not exhibit higher swelling ratios compared to the samples without this additive wherein in the case of the swelling performed in hemoglobin even worse sorption capacity was observed (3.80 g/g for unmodified sample and 3.39 g/g for sample with 2.5 mL of “cold starch”). This was probably caused by too little starch suspension used for the modification of hydrogels. The temperature of the starch suspension introduced into the hydrogel matrix may also be the cause of the swelling properties of such modified materials not increasing. Due to the fact that the starch suspension introduced into the reaction mixture was at room temperature, the molecules of starch did not dissolve in the solvent (distilled water) and the mixture added was only a suspension of starch in distilled water. Thus the starch inclusion was higher when this polysaccharide was introduced in the form of a hot solution than as a cold suspension. The introduction of a high temperature starch solution into the reaction mixture subjected to the photopolymerization process resulted in the increase in the values of swelling ratios calculated for all tested liquids (regardless of the volume of the introduced solution). Therefore, it may be concluded that such modified materials in the majority cases are appropriate for application in medicine, for example, in a form of wound dressings of which task is to inhibit heavy bleeding, because starch-modified hydrogels are able to absorb larger volumes of liquids compared to unmodified polymers.

### 3.6. Analysis of the Chemical Structure of Hydrogels Modified with Starch via FT-IR Spectroscopy

**Figure 9:**
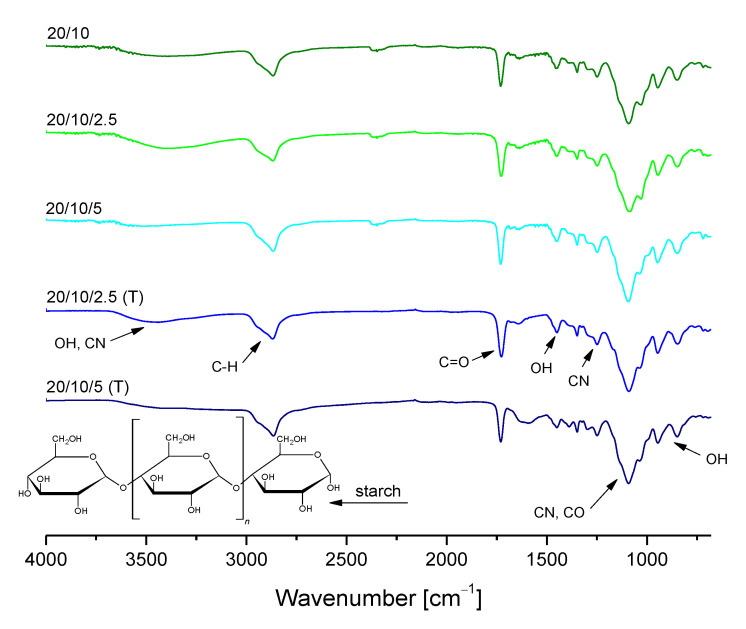
FT-IR spectra of hydrogels with starch compared to the unmodified materials.

Results of FT-IR spectroscopy of hydrogels containing starch are shown in Figure 9. Obtained FT-IR spectra are presented together with the FT-IR spectra of unmodified polymers to compare the structures of both materials.

Based on the FT-IR spectra of starch-modified hydrogel materials before and after modification it was observed that the material did not significantly change its chemical structure. On the spectra of modified polymers any new bands which could derive from the additive (starch) were not observed. On the other hand, a dependance between the intensity of bands from hydrogels with starch solution (“cold starch”) and starch suspension (“hot starch”) was observed. Bands deriving from samples modified with 2.5 mL of starch solution (“hot starch”) showed higher intensity than bands observed on the FT-IR spectra of samples containing starch solution prepared at room temperature (“cold starch”). The same conclusion may be drawn analyzing the bands deriving from samples modified with 5 mL of starch solutions. This may be caused by the fact that in the case of modification of hydrogels using “hot starch” a higher starch inclusion in obtained hydrogel matrices may be observed. This, in turn, is related to the larger number of hydrophilic groups specific for this polysaccharide and finally to the higher intensity of bands deriving from them. Moreover, functional groups present in the structure of starch duplicated with functional groups of the reagents forming the hydrogel matrix. This, in turn, resulted in the increase in the intensity of bands on the FT-IR spectra of samples modified with the high temperature starch solution. An example of such a functional group may be a carboxylic group which occurs both in the structure of starch and chitosan which is a main component of hydrogel matrix.

### 3.7. SEM Analysis of Hydrogels Modified with Starch

**Figure 10:**
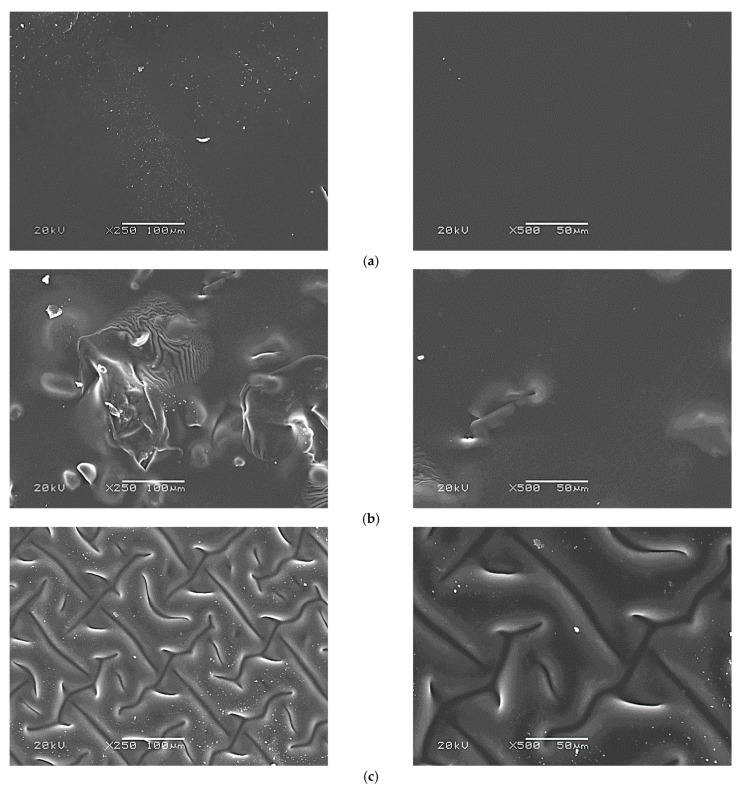
SEM images of hydrogel samples: 20/10 (**a**), 20/10/5 (**b**) and 20/10/5 (T) (**c**) (the analysis was performed using two magnifications: ×250 and ×500).

For every sample, SEM imaging was performed for four different areas (with two magnifications: ×250 and ×500). Here, in Figure 10, two representative images are presented while the other two for all samples are shown in Supplementary File (Figure S1).

On above-presented images a morphological structure of the surface of obtained hydrogel polymers may be observed. Each analyzed sample (20/10; 20/10/5; 20/10/5 (T)) was tested using two different magnifications, ×250 and ×500, and in four different areas. Unmodified hydrogel sample (a) showed a very homogeneous surface. Its surface is smooth and not corrugated. On the other hand, the surfaces of starch-modified materials are completely different. Hydrogel modified with cold starch suspension (b) showed visible inequalities on its surface which are heterogeneous. This, in turn, may confirm the addition of starch in a form of a solution—not a suspension—which caused such heterogeneous surface formation. It may also make the analysis of the sorption properties of such modified samples and their mechanical properties difficult and lead to the misleading conclusions (such material is characterized by heterogeneous and unrepeatable structure). However, in the case of the modification of hydrogels with hot starch solution (c) the surface of obtained polymers was homogeneously corrugated, and importantly, it was possible to prepare a repeatable structure corrugation.

Thus it may be concluded that the materials modified with cold starch suspension did not show repeatable characteristics of the morphological structure, therefore these polymers are not attractive in terms of future studies and potential biomedical applications.

### 3.8. Results of the Investigations on the Tensile Strength of Hydrogels Modified with Starch

**Figure 11:**
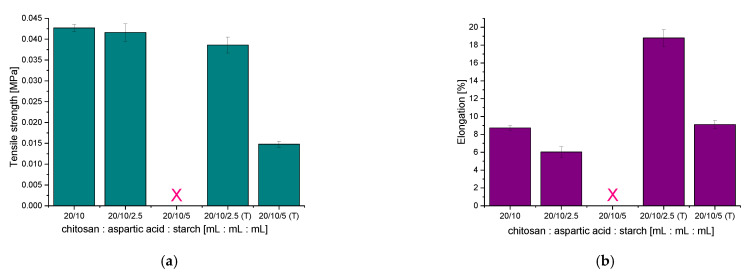
Tensile strength (**a**) and elongation (**b**) of hydrogels modified with starch.

Results of studies on the mechanical properties of chitosan/poly(aspartic acid)-based hydrogels containing starch including determining the elongation and the tensile strength under the tension applied are presented below in Figure 11.

Performed mechanical studies allowed to state that the materials modified with 2.5 mL of hot starch solution exhibited a high elasticity showing at the same time slightly worse tensile strength than unmodified hydrogels. The introduction of 2.5 mL of cold starch suspension into the hydrogel matrix resulted in the preparation of materials with comparable tensile strength to the unmodified material. Nonetheless, such modified hydrogels also showed lower elasticity (the resulted material was rigid) than a hydrogel without this polysaccharide. This is strictly related to the above-presented SEM images in which the highly heterogeneous structure of tested materials was observed. Starch present in the hydrogel matrix in the form of a cold suspension probably did not form any bonds with the polymer matrix which could improve its mechanical properties, i.e., elasticity.

Thus it may be concluded that the introduction of the hot starch solution into the hydrogel matrix resulted in the improvement of its mechanical properties—the improvement of such properties by this polysaccharide was also noticed by Almeida et al. [33]. Nonetheless, such an effect was observed only in the case of introduction of this additive in the amount of not larger than 2.5 mL. A larger amount of the starch solution in te hydrogel matrix resulted in worse mechanical properties of such modified hydrogels. This was probably caused by the presence of particles of amylose and amylopectin in the structure of starch which have a numerous amount of functional groups in their structures which may interact with groups of the reagents forming a hydrogel matrix. When too large an amount of this polysaccharide is introduced into the polymer, the mentioned interactions are too intense, additional chemical bonds between this additive and the matrix are formed and the structure of such material is more compact therefore its tensile strength decreases.

In the case of the hydrogel sample modified with 5 mL of the cold starch suspension, the analysis of its mechanical properties was not possible due to technical reasons—such a modified material, due to its rigidity and fragility, cannot be placed between the jaws of a texture analyzer.

## 4. Conclusions

- The volume ratio of chitosan and aspartic acid used for the synthesis of hydrogels affected their properties. It was proved that the larger the amount of aspartic acid compared to the amount of chitosan, the higher swelling ability of the hydrogels.
- Hydrogels based on chitosan and poly(aspartic acid) in a 2:1 volume ratio showed the most favorable properties, i.e., were elastic (9% elongation which was two times larger compared to the other samples), did not degrade after 30-day incubation in various simulated physiological liquids and did not show any structural changes as a result of such an incubation, and exhibited a relative biocompatibility towards tested liquids and similar swelling in each absorbed medium.
- Hydrogels modified with starch added as a hot solution showed higher sorption capacity and greater elasticity than polymers without this polysaccharide.
- Unmodified hydrogel sample showed smooth and very homogeneous surface. On the other hand, hydrogel modified with cold starch suspension had visible inequalities on its surface which were heterogeneous. Such heterogeneous surface formation of samples modified with cold starch suspension may make the analysis of their swelling and mechanical properties difficult and lead to misleading conclusions (due to the unrepeatable surface morphology of such materials). The surface of hydrogels modified with hot starch solution was homogeneously corrugated and it was possible to prepare a repeatable surface corrugation.
- The differences in the properties of materials modified with starch solutions prepared at different temperatures were caused by the fact that at hot temperature starch dissolved in water, and this additive was added in the form of a homogeneous solution. The application at room temperature resulted in the preparation of a starch suspension in which the starch particles were dispersed in the solvent and they were probably heterogeneously distributed in the polymer matrices. This, in turn, affected the morphology, elasticity and tensile strength of the hydrogels modified with such a suspension of this polysaccharide.

## Figures and Tables

**Table 1:** Compositions of chitosan/poly(aspartic acid)-based hydrogels.

Sample	Chitosan [mL]	Aspartic Acid [mL]	Crosslinking Agent ^(a)^ [mL]	Photoinitiator ^(b)^ [mL]
0/30	0	30	4.8	0.25
30/0	30	0		
15/15	15	15		
20/10	20	10		
10/20	10	20		

^(a)^ diacrylate poly(ethylene glycol). ^(b)^ 2-hydroxy-2-methylpropiophenone, Darocur 1173.

**Table 2:** Compositions of starch-modified hydrogels.

Sample	Chitosan [mL]	Aspartic Acid [mL]	“Cold Starch” [mL]	“Hot Starch” [mL]	Crosslinking Agent ^(a)^ [mL]	Photoinitiator ^(b)^ [mL]
20/10/2.5	20	10	2.5	-	4.8	0.25
20/10/5			5.0	-		
20/10/2.5 (T)			-	2.5		
20/10/5 (T)			-	5.0		

^(a)^ diacrylate poly(ethylene glycol). ^(b)^ 2-hydroxy-2-methylpropiophenone, Darocur 1173.

## Data Availability

The data presented in this study are available on request from the corresponding authors.

## Supplementary Materials

The following are available online at https://www.mdpi.com/article/10.3390/ma14164443/s1, Figure S1: SEM images of hydrogel samples: 20/10 (**a**), 20/10/5 (**b**) and 20/10/5 (T) (**c**) (the analysis was performed using two magnifications, i.e., ×250 and ×500).
