# Supplementary material for: Starch Solutions Prepared under Different Conditions as Modifiers of Chitosan/Poly(aspartic acid)-Based Hydrogels
Source: Materials (Basel). 2021 Aug 8;14(16):4443. doi: 10.3390/ma14164443 (PMC8399717; doi:10.3390/ma14164443)
Supplement: Supplementary file 1 [file materials-14-04443-s001.zip › materials-1329580-supplementary.pdf]

materials

Supplementary Materials

Starch Solutions Prepared under Different Conditions As Modifiers of Chitosan/Poly(aspartic Acid)-Based Hydrogels

Magdalena Głąb Anna Drabczyk, Sonia Kudłacik-Kramarczyk, Martin Duarte Guigou, Agnieszka Makara, Paweł Gajda, Josef Jampilek and Bożena Tyliszczak

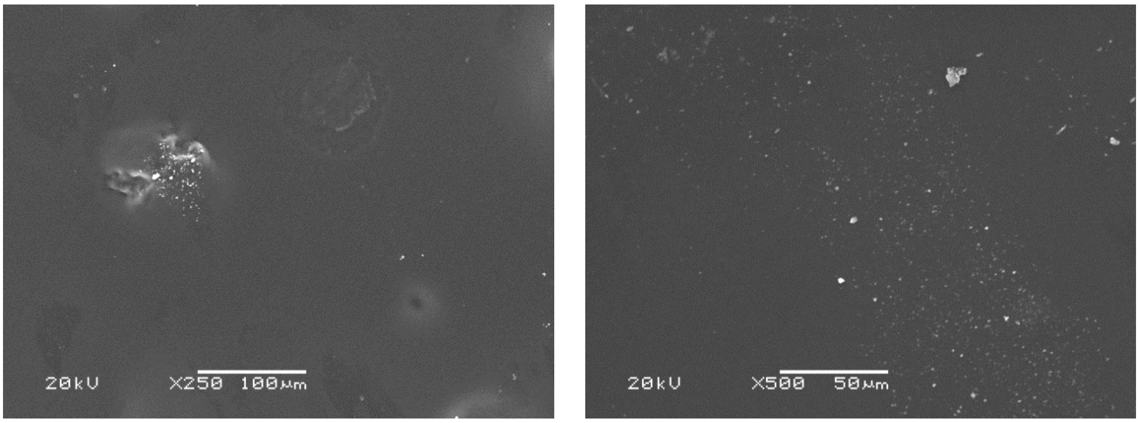

a)

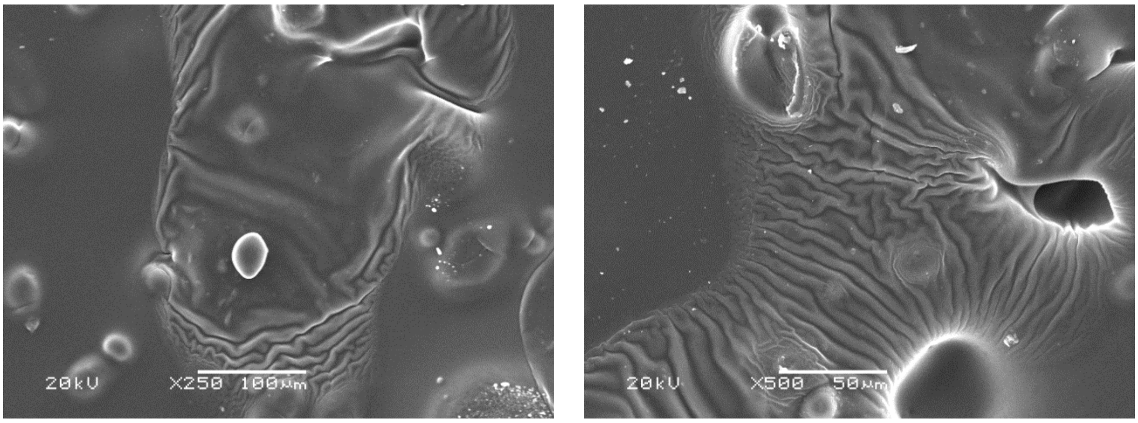

b)

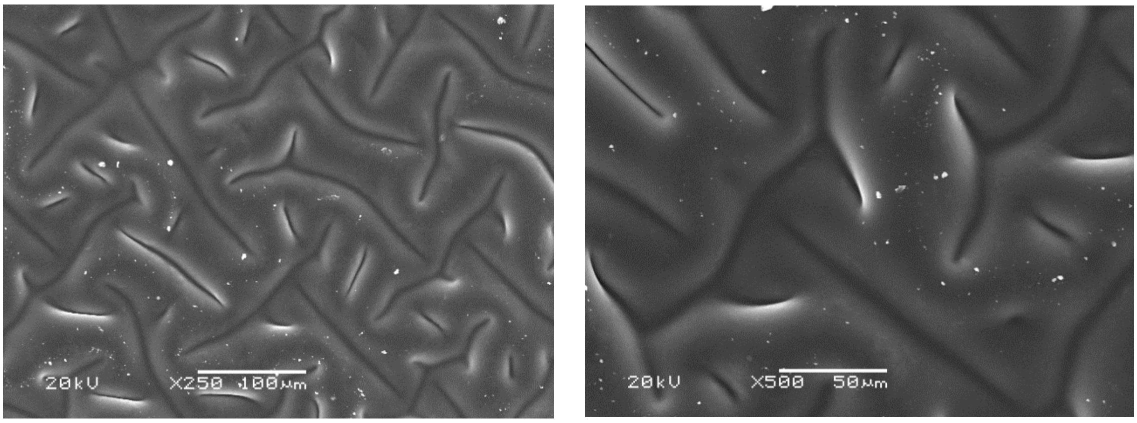

c)

Figure S1. SEM images of hydrogel samples: 20/10 (**a**), 20/10/5 (**b**) and 20/10/5 (T) (**c**) (the analysis was performed using two magnifications, i.e. ×250 and ×500).
